# ‘A landmark in psychiatric progress’? The role of evidence in the rise and fall of insulin coma therapy

**DOI:** 10.1177/0957154X211062538

**Published:** 2021-12-22

**Authors:** Robert Freudenthal, Joanna Moncrieff

**Affiliations:** University College London, UK

**Keywords:** History of psychiatry, insulin coma therapy, ‘The insulin myth’, insulin shock, treatment of schizophrenia

## Abstract

This paper examines the evidence behind the use and decline of insulin coma therapy as a treatment for schizophrenia and how this was viewed by the psychiatric profession. The paper demonstrates that, from the time of its introduction, there was considerable debate regarding the evidence for insulin treatment, and scepticism about its purported benefits. The randomized trials conducted in the 1950s were the result, rather than the origins, of this debate. Although insulin treatment was subsequently abandoned, it was still regarded as a historic moment in the modernization of psychiatry. Then, as now, evidence does not speak for itself, and insulin continued to be incorporated into the story of psychiatric progress even after it was shown to be ineffective.

## Introduction

Insulin coma therapy, a widely used treatment for people diagnosed with schizophrenia from the 1930s to the 1960s, is usually regarded as a useless and dangerous procedure sustained by indiscriminate medical enthusiasm, an example of ‘furor therapeuticus’ (Eisenberg, 2007; Jones, 2000). Critics of psychiatry suggest that ‘if insulin coma treatment is not a torture, nothing is’ and recall how it was ‘devastating’ and ‘painful’ for those who had to endure it (Frank, 2002).

Insulin coma therapy was eagerly embraced by the psychiatric profession in the mid-twentieth century, and promoted by leading psychiatrists of the time (Jones, 2000). Previous research has revealed how insulin treatment, along with other physical treatments like lobotomy and electro-convulsive therapy (ECT), helped in the profession’s struggle to establish its credibility as a genuine medical speciality (Moncrieff, 1999). The highly invasive nature of insulin coma therapy, the intensive medical monitoring, and specialized equipment needed to administer the treatment helped psychiatrists to persuade themselves and others that they were administering valuable, if dangerous, medical procedures (Doroshow, 2007). As psychiatrist and contemporary critic of insulin therapy Harold Bourne commented later, ‘it meant that psychiatrists had something to do. It made them feel like real doctors instead of just institutional attendants’ (cited in Doroshow, 2007: 243).

The consensus view is that insulin coma therapy, like other ineffective treatments, was discarded with the advent of modern research techniques and a new understanding of what constitutes proper and reliable evidence. Most authors credit the randomized controlled trials, published in the late 1950s, with having disproved the idea that insulin coma therapy was effective (Burns, 2019; James, 1992), especially the trial that compared it with a barbiturate-induced coma and found no difference in outcome (Ackner, Harris and Oldham, 1957). For some, it marks the start of the movement towards ‘evidence based medicine’ because it was the first therapy to be rejected on the basis of randomized controlled trials (Andrews, Briggs, Porter, Tucker and Waddington, 1997; Burns, 2019). Others have described how insulin coma therapy was, in its time, seen as an efficacious and scientific treatment for mental illness (Doroshow, 2007), suggesting that today’s view of evidence and what qualifies as ‘scientific’ is quite different from that of the recent past. Most authors assume that, in its heyday, insulin coma therapy was uncritically accepted as an effective treatment (Jones, 2000).

Against this view, psychiatrist Michael Shepherd argued that evidence had nothing to do with the decline of insulin coma therapy. He suggested that psychiatrists simply switched away from it to the use of antipsychotic medication for the treatment of schizophrenia, not as a result of evidence, but ‘like a large shoal of fish . . . to follow the lights of the more fashionable pharmacotherapy of schizophrenia’ (Shepherd, 1994: 93). Others have argued that the antipsychotic drugs introduced in the 1950s became the preferred treatment because they were simpler to administer and cheaper than cumbersome procedures such as insulin therapy and ECT (Healy, 2002: 55–6). Research suggests that at the time the drugs were regarded as the natural successors of the physical treatment procedures, such as insulin coma therapy, and not as a break from the past (Moncrieff, 1999). As such, the new drugs inherited the enthusiasm that had been attached to the physical treatments.

In the current project, we suggest that neither of these positions captures the full reality of the time. We reveal how there was a significant debate about the effectiveness of insulin coma therapy prior to the publication of the randomized trials, and much of it concerned the quality of the existing research and was conducted in recognizably modern terms. Whereas Doroshow (2007) argues that insulin coma therapy was regarded as scientific by the standards of the time, our research shows this was not universally true. Some psychiatrists undoubtedly saw it this way, but, from its early days, some felt the evidence supporting it was inadequate. The merits of insulin coma therapy were, it seems, a divisive issue – the procedure certainly had its champions, but it also had detractors. Harold Bourne’s famous paper ‘The insulin myth’ (1953), which denounced the treatment as a fraud, is a symptom rather than the origin of this debate. The fact that several randomized trials were set up to evaluate the procedure in the 1950s also suggests there was already significant doubt about its value.

The current project aims to explore the relationship between insulin coma therapy and scientific evidence in the period of its use and during its decline. We explore the nature of the evidence and how it was regarded by psychiatrists at the time, and also the role that the evidence played in the abandonment of the treatment. We look, in particular, at whether it was rejected on the basis of new and modern evidence about its effects.

Sources include academic papers retrieved by searching PubMed with the search terms ‘insulin’ and ‘schizophrenia’, restricted to a date of publication from 1930 to 1965; succeeding editions of the principal British psychiatric textbooks; and audio recordings in the British Library Archive of interviews with psychiatrists and patients who had experienced the insulin era.

## What was insulin coma therapy?

In 1933, the Austrian psychiatrist Manfred Sakel reported to the Medical Society of Vienna the positive results from his experiments with giving insulin to people with schizophrenia (Sakel, 1937). Insulin coma therapy was rapidly adopted across Europe and North America, and by 1939 was being promoted by governmental public health bodies, and favourably reported in the mainstream media (Adams, 2014). It continued to be used until the early 1960s, but by the late 1960s the treatment had largely been abandoned, although there are some reports that it continued to be used in Australia as late as 1974, and in China in 1985 (Kaplan, 2013).

Insulin coma therapy involved giving patients a dose of insulin large enough to induce a hypoglycaemic coma. The exact procedure varied from unit to unit, but patients were maintained in an unconscious or semi-conscious state for approximately three hours and then the coma was terminated by giving the individual some light food or an injection of glucose. After being given the procedure in the morning, the patient would be expected to participate in occupational therapy and recreational activities in the afternoon (Mayer-Gross, Slater and Roth, 1954: 552). This treatment would then be given for approximately 30 consecutive days, apart from Sundays.

Insulin coma units were tightly controlled and intensively staffed, with an environment similar to that of an operating room (Doroshow, 2007). During the course of the procedure, regular physical and neurological tests would be carried out and, if there were concerns about the patient’s safety, staff could terminate the coma early by giving a sugary drink via a nasogastric tube, or by an intravenous injection of 33 per cent glucose solution. Nevertheless, the mortality rate was high, with contemporary reports estimating it to be between 0.5 per cent (Freudenberg, 1947) and 4.5 per cent (Ebaugh, 1943).

A graphic description of insulin coma therapy was published in the *British Medical Journal* in 1937:

within about half an hour he [the patient] becomes restless, tossing from side to side, at first moaning and later crying out . . . furor may set in. He leaps up in bed, staring and crying out aloud. He throws himself about violently and has the appearance of maniacally resisting a great fear. He froths at the mouth and his pupils dilate. He may beat his head and hands and feet frantically . . . coma follows and the patient lies shrunken into his bed, profoundly collapsed, with a diminished pulse . . . he salivates profusely, and must be laid on his side to allow the saliva to run out . . . from time to time he may be convulsed. Occasionally a true epileptic fit may be seen with epileptic cry, a tonic stage with cyanosis, and a clonic stage. (Larkin, 1937: 746)

This account, by EH Larkin of the West Ham Mental Hospital in London, is echoed by British Library Archive interviews with two patients. They described the procedure as ‘unpleasant’ and ‘dangerous’ (C905A/09/01, 1999) and noted how ‘people felt liberated when they didn’t have to have it any more’ (C905/01/10 C1, 1999). A psychiatrist who worked at the time described it as ‘a very drastic treatment . . . [the] patient [was] brought to the jaws of death many times’ (C512/42/01-02, 1991).

## The rise of insulin coma therapy, 1937–53

The published literature from this period suggested there was growing excitement about the use of insulin coma therapy as a treatment option for schizophrenia, alongside a debate about whether it was, in fact, effective or worthwhile. Authors of academic papers published in medical and psychiatric journals were mostly psychiatrists, and most were enthusiastic about the new treatment option. From the late 1930s, papers referred to insulin coma therapy being widely used, and several articles called for its use to be expanded. An editorial published in the *British Medical Journal* in 1938, for example, argued that the treatment was most effective if given early in the course of the condition diagnosed as schizophrenia, and suggested the need for additional training to enable medical staff to recognize early stages of the disorder so that more people could receive the treatment promptly (Anon., 1938).

Larkin’s (1937) paper was aimed at persuading his colleagues around the UK of the feasibility of setting up an insulin coma programme in their local hospitals. He described his own results with the treatment as ‘fully justifying what was initially regarded as in the nature of an experiment’ (p. 745). He also noted the importance of building up enthusiasm among nursing colleagues, and of training them carefully to manage the potential complications of the procedure, including not over-reacting when the patient’s appearance was ‘very alarming’ (p. 746).

Many papers claimed to show evidence of insulin’s effectiveness based on data from a series of patients who had undergone the procedure. The numbers of patients described in these reports varied from 5 to over 100 (Anon., 1938). In Sakel’s first published paper on insulin treatment for schizophrenia, he claimed that 88 per cent of his first group of over 100 patients with a recent onset of schizophrenia showed a good degree of improvement following insulin coma therapy, with 70 per cent making a full recovery (Sakel, 1937). He suggested these rates were far in excess of what would be expected from the natural history of the condition. The following year, Easton conducted a comparative study in Canada, and reported that 65 per cent of patients who received insulin treatment recovered, compared with only 40 per cent of those who were treated in the same unit but did not receive insulin (Easton, 1938). He concluded that ‘in insulin we have a drug by means of which we can alter the clinical picture in schizophrenia’ (p. 236). Johnson (1939) published a large study, also in Canada, involving data from 108 patients who had undergone insulin coma therapy between 1937 and 1938, and 322 patients treated at the same hospital between 1932 and 1936 before insulin coma therapy was introduced: 82 per cent of patients who had insulin coma therapy were discharged versus 47 per cent of patients from the earlier period. He concluded that ‘insulin shock treatment appears definitely to increase the patient’s prospects of being benefited by hospital care’ (p. 66). McConnell (1945) published a similar paper, citing an 88 per cent discharge rate among insulin-treated patients in a British unit, compared to 48 per cent among people treated before insulin was instituted.

Other articles, however, reported no evidence that insulin was beneficial. Libertson (1941) described results of treatment of 330 patients in a hospital in New York State, USA, and found no difference in rates of improvement or recovery between those who were treated with insulin and those who were not. Teitelbaum et al. (1946) reported the results of a controlled trial in the USA comparing insulin coma therapy, a barbiturate-induced coma, a placebo procedure and general care. They found little difference between the procedures and concluded that improvement was independent of the mode of treatment. Palmer, Riepenhoff and Hanahan (1950) described the outcome of a series of 393 patients in the USA who had received insulin coma therapy: only 34 per cent were discharged, and only 6 per cent showed a full recovery at follow-up, which was 3.3 years later, on average. The authors concluded that insulin was ‘certainly not sufficient as a sole form of treatment in schizophrenia’ (p. 925).

Further evidence suggests there was an active debate about the effectiveness and value of insulin coma therapy at this time. At a meeting at the Royal Society of Medicine in 1938, Dr Freudenberg, himself a keen proponent of insulin coma therapy (Jones 2000), and his colleagues referred to critics of the procedure: ‘we feel that in some quarters there is a tendency to magnify the dangers of insulin therapy’ (James, Freudenberg and Cannon, 1938: 578). Concerns were raised about the methodology of insulin research, with critics pointing to the poor quality of the studies that were used to demonstrate its effectiveness (Libertson, 1941). Others noted that the diagnostic criteria for schizophrenia varied, and therefore it was difficult to compare treatment outcomes in different groups (Easton, 1938). In a reflection on more than 10 years of insulin therapy, Freudenberg (1947) highlighted ‘the difficulties in evaluating the effectiveness of this method’ (p. 9), including the problem of identifying appropriate control or comparator groups, variations in the quality and duration of improvement, and the fact that the ‘enthusiastic therapist’ (p. 10) might discharge patients earlier than they would have done otherwise, thereby skewing any results presented in terms of discharge. Among his recommendations for conducting the procedure, he noted how it was ‘generally agreed’ that the response to insulin was better among people who had a shorter duration of illness or a ‘good prognosis without any special treatment’ (p. 19), a point that was highlighted by later critics.

These criticisms continued into the early 1950s, when prominent supporters of the treatment commented that it was ‘still in need of justification’ (Mayer-Gross, 1951: 132). Yet despite the lack of agreement regarding its evidence base, it is referred to as being widely used by the early 1950s (Reznick and Arnett, 1951).

The principle British textbook of this time, Henderson and Gillepsie’s *A Text-book of Psychiatry*, reflected the debate about the efficacy and value of insulin coma therapy by presenting apparently contradictory views, suggesting a conflicted attitude towards the procedure. For example, the fifth edition (Henderson and Gillespie, 1940) commented that insulin had the potential to lead ‘to the complete abolition of all schizophrenic symptoms’ (p. 315), but also expressed scepticism about the data. The book pointed out that Sakel’s figures were derived from patients seen in his outpatient clinic, and ‘belonged, therefore, to an earlier and more recoverable group than is indicated by admission to a mental hospital’ (p. 316). The authors came to the conclusion that the effects of insulin coma therapy are ‘difficult to estimate’ (p. 317), but did seem to think that it provided a potential avenue for effective treatment if the intervention were to be further developed and researched.

The sixth edition of the textbook (Henderson and Gillespie, 1944) repeated doubts about the evidence, stating that ‘there is not so much to choose between these figures, before and after insulin, as may appear on first inspection’, and they made a plea for further studies with more rigorous methodology: ‘the only sound type of experiment is one which takes two closely similar series of cases . . . and treats one series with insulin and one without’ (p. 375). Despite this, the authors still considered insulin coma therapy to represent a ‘considerable advance’, stating there was a ‘quantitative relationship between dosage and clinical effect’ (p. 374). They also hypothesized that the treatment was ‘acting in some way on the nervous pathways which is so important’ (p. 375).

The seventh edition also included a discussion about the limitations of the evidence, concluding that ‘the efficacy of insulin shock therapy is still, therefore, somewhat difficult to estimate’ (Henderson and Gillespie, 1950: 417). The authors considered that ‘insulin is capable of shortening the course of schizophrenic illness’ but ‘what is not yet certain is whether it does more than this’ (p. 415). They still referred to the idea that the procedure was acting on ‘nervous pathways’ (p. 414).

Interestingly, despite the risks associated with insulin coma therapy, and Freudenberg’s claim that critics ‘magnified its dangers’, little attention was paid to the harmful effects in any of the written material examined. Henderson and Gillespie (1950: 408) acknowledged that the procedure could be risky in ‘insufficiently skilled hands’, but reassured readers that ‘in practised hands the mortality is about one per cent’. There was a brief description of some ‘complications’, including nephritis and pulmonary oedema, but many events that would be considered harmful today, such as epileptic fits and protracted coma, were regarded as part of the therapeutic process.

## The decline of insulin, 1953–65

‘The insulin myth’ was published in *The Lancet* in 1953 (Bourne, 1953). The author, Harold Bourne, was a junior doctor at the time, and the paper involves a long and substantial presentation of the inadequacy of the evidence in support of insulin and of the superiority of ECT. Although later historical accounts frequently refer to the paper as being a landmark publication in shifting psychiatric opinion away from the widespread use of insulin coma therapy (Pimm, 2014), Bourne’s arguments had already been expressed in the published literature. Like earlier critics, he highlighted how many studies of insulin therapy involved small numbers of patients, had no proper comparator group, were not blinded, and that there were no standardized definitions of schizophrenia itself or of recovery. As such, Bourne (1953) represented a debate that was already in existence, which is supported by the fact that *The Lancet* was prepared to publish a detailed, five-page article by this unknown junior doctor. However, he made the argument in more detail, and more forcefully than his predecessors. He also addressed the idea that insulin might target the putative pathology of schizophrenia, an idea he reported as being promoted in educational material available at the time.

‘Insulin offers the schizophrenic no long-term benefits’, he states and:

at best it hastens improvements that would occur anyhow with simple care in hospital, and which could be accelerated at least as well with E.C.T., and there is no evidence that this uncertain benefit is attributable to the insulin itself and not to the insulin-treatment situation. If the latter is the operative factor, its effects could surely be reproduced by some more rational and less hazardous means. (p. 967)

He concludes ‘there is no proof of any specific therapeutic effect’ (p. 968).

Bourne’s paper prompted an extensive discussion in the ‘letters’ section of *The Lancet*, further illuminating how the debate about insulin coma therapy was already taking place. Of the 14 responses, 9 letters were broadly critical of Bourne’s arguments and supportive of insulin. Several letter-writers offered their own clinical experience and anecdotes of patients improving after receiving the therapy (Cook, 1954; Gibson, 1953; Rees, 1953). Prominent advocates of insulin coma therapy William Sargant and William Mayer-Gross were also among the respondents. Sargant (1953) criticized Bourne for selecting his evidence and omitting studies that showed positive effects. He cited how opinions expressed at the International Congress of Psychiatry in 1950 confirmed that insulin was the ‘generally accepted treatment for this condition’). However, he also suggested that ‘insulin is certainly not a specific treatment for schizophrenia, but the best we have available at present for many patients in the early stages’. Mayer-Gross (1953) acknowledged that the data supporting the treatment had been ‘severely and authoritatively criticised’. ‘Nevertheless,’ he continued, ‘most clinicians have refused to abandon the method and others have even resumed it during the last few years’, because, he argued, ‘it is the only treatment that holds out some hope of shortening and relieving schizophrenia’. Several other respondents felt the poor prognosis of schizophrenia and hopelessness of recovery justified the treatment, as ‘at present the best we can offer’ (Friedman, 1953).

Five letters supported Bourne, with some writers describing insulin coma therapy as a ‘prolonged and dangerous form of therapy’ (Jarvie, 1953) and others presenting further data illustrating the ineffectiveness of the procedure (e.g. Davis, 1953; Lehoczky and Lehoczky, 1954).

The most forthright of Bourne’s defenders, psychiatrist Richard Hunter from Guy’s Hospital, London, suggested that Bourne’s ‘commentary on the use of insulin in schizophrenia demonstrates how easily irrational therapies are introduced, how readily they are accepted, and how hard they die’ (Hunter, 1953). He went further than Bourne, who advocated for ECT, in criticizing physical methods of treatment in general, suggesting that:

Further, physical methods of treatment themselves produce an abnormal picture, characterised by loss of spontaneity, loss of memory for recent events, diminution or loss of overt signs and symptoms of illness, and increased socialisation and hence easier management. These are taken as evidence of therapeutic success, and little attention is paid to the price in concomitant over-all reduction of mental function. (p. 1152)

The letter prompted Hunter’s colleague, David Stafford-Clark (1953), to write to *The Lancet* distancing himself and Guy’s Hospital from Hunter’s views, and reassuring readers that insulin was not an ‘irrational therapy’ and that it remained a ‘standard procedure’ at Guy’s Hospital. To rebut Hunter’s suggestions about how insulin affected people, he cited papers suggesting insulin therapy worked by increasing blood oxygen saturation.

The other paper frequently referred to as a ‘landmark’ in the decline of insulin coma therapy (Burns, 2019) is the report of a randomized controlled trial carried out by Brian Ackner and colleagues at the Maudsley, Bethlem and Cane Hill Hospitals in London and Surrey and published in 1957. The study compared insulin coma therapy with a barbiturate-induced coma using random allocation and was one of the first randomized controlled trials in psychiatry (Ackner et al., 1957). Despite being so influential, it only involved 50 patients. The results showed no difference between the two procedures, but the authors were reluctant to declare that insulin was ineffective, suggesting only that ‘no conclusion can be drawn about the therapeutic value of the coma regime, but the results suggest that insulin is not the specific therapeutic agent’ (p. 611).

In the previous year, results of a randomized trial comparing insulin coma therapy with a new drug, chlorpromazine, were published. This study showed a small advantage for patients treated with chlorpromazine, although the difference in outcomes was not statistically significant, and the authors cautiously referred to it as ‘inconclusive evidence’ (Boardman, Lomas and Markowe, 1956: 490). Nevertheless, they proposed that chlorpromazine should be the ‘treatment of choice’ for people with schizophrenia, and insulin should be reserved for people in whom this did not work. They also mentioned the disadvantages of insulin treatment as including the ‘greater danger and more unpleasantness for the patients and greater strain on the nurses’.

Both of these trial reports opened with extensive discussions about the methodological drawbacks of previous research, confirming that a debate about this was already taking place. Boardman et al. (1956: 487) referred to the ‘dissident voices’ who questioned insulin’s value. Although these studies themselves would fall short in many ways by modern standards, they did involve randomization, blinded assessment of outcome and crude standardized outcome measures. Most importantly, this meant they were able to compare groups of patients who were reasonably similar in outlook.

Two randomized trials were published subsequently, both comparing insulin coma therapy with chlorpromazine and one also involving ECT. One found no difference between insulin and chlorpromazine and concluded that ‘no evidence has been deduced that either therapy has altered the basic schizophrenic process’ (Fink, Shaw, Gross and Coleman, 1958: 1850). Only one of the four randomized trials found an advantage for insulin coma therapy. This study reported higher relapse rates among people treated with chlorpromazine compared to those given insulin or ECT, but the numbers of patients involved were small (between 15 and 18 in each treatment arm) (Baker, Game and Thorpe, 1958).

In the ninth edition of their textbook, Henderson and Gillespie (1962) cited the Ackner study as providing evidence that insulin coma therapy did not have any specific effect on schizophrenia, and recommended that the treatment should no longer be used. However, they continued to view it as having been a positive development for psychiatry, and considered many patients to have benefited from it, arguing for the intensive medical and nursing attention that went alongside the treatment to continue (p. 347). In the last edition of the textbook, the authors referred to insulin coma therapy as ‘a landmark in psychiatric progress’ (Henderson and Gillespie, 1969: 458), although they also implied that it was adopted too readily without a proper evidence base. When discussing their concerns about the new drug treatment of schizophrenia, which they also felt lacked evidence or a clear rationale, they suggested that psychiatrists were repeating past ‘mistakes’, ‘using these new therapeutic agents too enthusiastically, too indiscriminately and frequently naively’ (p. 475). The contradictory attitudes that run through all the editions of this textbook suggest, perhaps, that despite their scepticism, the authors could not help but see something appealing and progressive in a highly technical, scientific-looking procedure. They viewed modern drug treatments as analogous to physical procedures, such as insulin coma therapy, being desirable, but not specific. In the 1969 edition, after the widespread adoption of the new drugs, they concluded there was still ‘no specific and effective treatment for schizophrenia’ (p. 483).

Mayer-Gross et al. were more defensive of insulin coma therapy in the early editions of their textbook *Clinical Psychiatry*, which is understandable since William Mayer-Gross was a well-known advocate of the treatment and Elliot Slater was a prominent biological psychiatrist who co-authored a textbook on physical treatments in psychiatry with William Sargent, another vociferous promoter of physical approaches (Jones, 2000). In the first edition of *Clinical Psychiatry*, (Mayer-Gross et al., 1954), the authors acknowledged that insulin coma therapy had ‘been the target of much criticism’ (p. 552), but considered it to be the ‘only effective method of treating early schizophrenia’ (p. 552), claiming that ‘if applied within the first year of the illness it more than doubles the number of remissions that can be expected to occur spontaneously’ (p. 552). In the second edition, they still considered it to be ‘one of the effective methods of treating early schizophrenia’ (Mayer-Gross et al., 1960: 604). They were not convinced that the Ackner study demonstrated it was ineffective, drawing instead, like Sargant, the oblique conclusion that the trial ‘could only demonstrate that it is not a specific remedy for schizophrenia, a claim never seriously made’ (p. 605). It is difficult to accept, however, that ‘non-specific’ effects could possibly have justified the use of such a dangerous and cumbersome procedure, and like Sargant, in his response to Bourne, the authors do not explain what they meant exactly or the implications of such a conclusion.

The authors of *Clinical Psychiatry* acknowledged that the new drug treatments were simpler and ‘dependable’, but like Henderson and Gillespie, they did not view them as ‘specific’ nor as marking a radical departure from previous psychiatric interventions. Rather, they echoed Henderson and Gillespie in suggesting that insulin coma therapy had been the landmark event in the development of psychiatric treatment. However, their insistence that ‘it shows little insight into its practice and mode of action . . . to compare it [insulin coma therapy] with the revolving chair or the diving bridge and other shock methods used by psychiatrists early in the nineteenth century’ (Mayer-Gross et al., 1960: 603) suggests that not everyone agreed with them.

Other influential psychiatrists also continued to defend insulin coma therapy. In 1958, Leslie Cook, President of the Medico-Psychological Association, discussed physical treatments in his ‘presidential address’ published in the organization’s journal. He acknowledged that insulin coma therapy had ‘always been controversial’, but continued to promote the view that individual clinical experience supersedes other concerns, and that insulin coma therapy and other physical treatments should be continued – ‘nobody likes the physical treatments in psychiatry: they are cumbersome, often lengthy, and have their dangers, but when selected and administered with thoughtfulness and care, their failures and occasional disasters are infinitesimal compared with the benefits they bring’ (Cook, 1958: 936). By the mid-1960s, several authors bemoaned the replacement of insulin by drug treatment (e.g. Freund, 1962), and some repeated the notion that despite the fact that it had no specific effect, insulin coma therapy, along with other physical treatments, had constituted a ‘revolutionary change’ in psychiatric treatment (Kelly and Sargant, 1965: 147).

In an interview, a psychiatrist confirmed that there were two camps with respect to insulin coma therapy by this time: ‘there was a great division between the deep insulin coma therapy boys who were very attached to their treatment and the people simply giving Largactil [chlorpromazine] which was very easy to give and claimed equally good results’ (British Library Archive, C512/42/01-02, 1991). He also described his reluctance to reject the therapy, admitting that he was a ‘committed deep insulin coma man’ and how the treatment had been regarded as ‘very prestigious’. Eventually, however, he ‘had to acknowledge that the effects were non-specific and you could get just as good results from chlorpromazine’.

## Mechanisms of action

Evidence in medicine traditionally includes an understanding of the pathological mechanisms involved in disease and the mechanism of action of potential treatments. It is notable, therefore, that the majority of the literature examined made no reference to possible mechanisms of action of insulin coma therapy. Most of the authors who praised the treatment, and those that questioned it, based their views on how it affected patients’ presentations, with little apparent concern about, or even interest in, how this might be achieved.

Among the small number of academic papers that did discuss mechanisms of action, the majority proffered a biological account of its action, while a small number proposed psychoanalytic explanations. The most commonly cited theories included the idea that insulin coma therapy offered an assault on, or interruption of, abnormal neurological pathways, and therefore facilitated a return to a ‘normal’ state. This theory originated from Sakel’s suggestion that insulin causes an ‘annihilating assault’ on the cell (Easton, 1938: 233; Ingham, 1937: 32), which on recovery from the assault allows the re-establishment of ‘older’, and presumably less psychosis-inducing, pathways (Borenz, Schuster and Downey, 1949; Easton, 1938). In 1958, Sakel explained that the treatment worked by selectively killing diseased brain cells, allowing ‘dormant ones to come into action in their stead’ (Sakel, 1958: 334). He likened the process to ‘fine microscopic surgery’. Animal and post-mortem studies published at the time demonstrated that insulin coma therapy was associated with extensive and irreversible brain cell destruction, whose extent correlated with the number of comas received, but not that there was anything abnormal about the brain to begin with (Kalinowsky and Hoch, 1950: 81–3; Sadler, 1953: 1042). There was also no evidence that this effect was beneficial, although some suggest, like Hunter, that it may have made patients quieter and more manageable (Whitaker, 2002: 91).

Some psychiatrists likened the effects of insulin coma therapy to lobotomy, which was also widely accepted as beneficial and believed to work in the same way. In 1956, an article in the *American Journal of Psychiatry* proposed that insulin coma therapy ‘worked’ by bringing about irreversible changes to the frontal and temporal lobes of the brain in what amounted to a ‘physiological lobotomy’ (Parfitt, 1956: 247). Hunter’s (1953) suggestion that all physical treatments produce an abnormal state of mind characterized by reduced mental capacity, also drew parallels between insulin coma therapy and lobotomy, but Hunter emphasized the abnormality of the states induced by these procedures rather than their curative potential.

The idea that insulin coma therapy was effective due to ‘endocrinological factors’ (Freudenberg, 1952: 441) was another popular suggestion, with pituitary hormones, sex hormones and cortisol, and carbohydrate metabolism all suggested as possible candidates for involvement in its mechanism of action (Freudenberg, 1952; Gottfried, Batelson and Pincus, 1953; Merlis and Michael, 1954).

Despite the relative lack of attention to the mechanism of action in research literature, up until the 1950s, there appeared to be an implicit understanding that insulin coma therapy was ‘acting in some way on the nervous pathways’, a view that Henderson and Gillespie (1944: 375) cited as being ‘so important’. As late as 1960, an article described insulin as ‘Sakel’s decisive step from a purely symptomatic to a curative treatment’ (Gilmore and Braun, 1960: 1626). Bourne (1953: 964) also referred to the ‘general opinion (as stated, for example, in a refresher course by Sargant and Slater in 1951) that insulin-coma therapy counteracts the schizophrenic process in some specific manner’. In defending insulin against Bourne’s attack, Stafford-Clark (1953) cited data on insulin increasing oxygen levels in the blood as evidence of a mechanism of action (Stafford-Clark, 1953). The fact that prominent proponents of the procedure, such as Sargant and Meyer-Gross, were keen to deny that they had ever claimed it was ‘specific’, suggests that this was, indeed, part of the existing rationale for treatment at which the critics took aim.

Biological theories about the mechanism of action of insulin, therefore, were mostly implicit but important, and the lack of a consensus about a mechanism of action was part of the critique of insulin coma therapy that gained momentum in the 1950s.

Psychoanalytic explanations highlighted the non-specific aspects of insulin treatment. They focused particularly on the role of the fear induced by the procedure. It was proposed that fear created a significant group attachment for the patients receiving the treatment together on the same unit, and an intense relationship between the patient and the staff, both of which might be therapeutic (Freudenberg, 1947; Gottlieb and Huston, 1951). The idea that insulin coma treatment induced a state of dependency and regression that replaced the patient’s need for regression through psychosis was also put forward (West, Bond, Shurley and Meyers, 1955), and some linked this state to the effects of brain trauma (Palisa and Flach, 1938).

## Discussion

Although ‘the prolonged use and advocacy of ineffective treatments is a commonplace of medical history and current practice’, as Jones (2000: 149) puts it, insulin coma therapy is one of the most notorious recent examples of this tendency. It was an ineffective, unpleasant and highly dangerous procedure, but it masqueraded as a sophisticated medical technique. Many psychiatrists, including leading figures of the day, were convinced it was beneficial and promoted its use.

Most previous accounts suggest that insulin therapy was viewed as a scientific innovation by the psychiatric profession of the time and that it was rejected only when modern research practices were introduced which demonstrated it to be ineffective. Our research demonstrates that insulin coma therapy was not universally accepted, however, and that, from its introduction in the 1930s, there was scepticism about its value as well as criticism of the scientific basis for its use.

While there was debate about the value of insulin coma therapy, there was near universal acceptance that psychiatric treatments should be based on scientific evidence. Both the proponents of insulin coma therapy and those who were more sceptical used published research to support their claims. Supporters of the technique cited quantitative summaries of patient outcomes and comparisons with other groups of patients. Critics highlighted the methodological limitations of these studies, including the lack of comparable control groups and the influence of investigator enthusiasm – concerns that reflect modern debates in evidence-based medicine.

The idea that the 1957 Ackner study was a watershed moment in the history of psychiatry, which heralded a new era of evidence-based effective treatments, and a departure from unpleasant and unscientific treatments of the past, is not supported by our analysis, and appears to be a modern interpretation of events. The randomized trials that demonstrated the ineffectiveness of insulin coma therapy were the result of a preceding debate about the quality of the evidence, and not the start of it. Nor did these trials convincingly demonstrate the superiority of the new drugs. The drug therapy that superseded insulin was regarded as a continuation of the type of modern medical intervention that insulin was seen as representing, even by those who had never been convinced of its effectiveness. Insulin therapy was not rejected by its advocates, nor by many sceptics; it was just quietly forgotten. Although the evidence was widely debated, other pressures ultimately dictated the direction of clinical practice, as Shepherd (1994) pointed out. The perceived medical credentials of the procedure outweighed concerns about its safety or effectiveness, until an acceptable medical alternative was available.

The story of insulin coma therapy illustrates how evidence can be interpreted in different ways. Even before the antipsychiatry movement, which deconstructed the idea that mental disorders are biological conditions which respond to targeted physical interventions (Szasz, 1970), there was already debate and disagreement about the nature of psychiatric treatment and the evidence that was supposed to support it.

It is striking that the majority of literature on insulin coma therapy focused on how it affected patients’ superficial presentation, and neglected questions about how it achieved its effects, what impact it had on the body and brain, the harms and dangers of the treatment and the nature of the patient experience. Nevertheless, there was an implicit understanding that it worked by targeting the underlying disease of schizophrenia in some way. This is similar to the present situation in relation to psychiatric drugs. Drugs such as antidepressants and antipsychotics (as they are now known) have not been demonstrated to act on pathological processes assumed to underpin depression, schizophrenia or psychosis, but are implicitly understood to do so and sometimes explicitly presented in this way (Moncrieff, 2008). Like insulin coma therapy, therefore, modern drugs are used on the basis of observed changes in behaviour and reported thoughts and feelings, without a consensus on how they might induce these effects and how they modify normal biological processes and brain functions. Critics suggest that their adverse effects are neglected and that they work by restricting normal mental capacities (Breggin, 2008), which is reminiscent of Hunter’s (1953) description of the effects of physical treatments as a whole.

Appreciating that the evidence for a widely accepted treatment like insulin coma therapy was debated in its own time, and interpreted in different ways, helps to inform current debates about the evidence for various common psychiatric treatments, such as antidepressants (Jakobsen, Gluud and Kirsch, 2019). The fact that even sceptics viewed insulin treatment as a ‘landmark in psychiatric progress’, despite not believing that it actually worked, suggests that ultimately non-scientific factors may determine what treatments are endorsed. Insulin coma therapy was an important part of the story of psychiatric progress. As Bourne (1953) suggested, it helped psychiatrists to believe in themselves as doctors, and it provided something medical to offer when there were few specifically medical alternatives. Then, as now, scientific evidence did not speak for itself, and did not necessarily have the last word.

## References

[bibr1-0957154X211062538] British Library Archive, London: Sound and Moving Image interviews: C512/42/01-02 (1991) On mental health testimony; C905A/09/01 (1999); C905/01/10 C1 (1999).

